# Hydrostatic Pressure Regulates the Volume, Aggregation and Chondrogenic Differentiation of Bone Marrow Derived Stromal Cells

**DOI:** 10.3389/fbioe.2020.619914

**Published:** 2021-01-15

**Authors:** Paola Aprile, Daniel J. Kelly

**Affiliations:** ^1^Trinity Centre for Biomedical Engineering, Trinity Biomedical Sciences Institute, Trinity College Dublin, Dublin, Ireland; ^2^Department of Mechanical, Manufacturing and Biomedical Engineering, Trinity College Dublin, Dublin, Ireland; ^3^Advanced Materials and Bioengineering Research Centre, Royal College of Surgeons in Ireland and Trinity College Dublin, Dublin, Ireland

**Keywords:** HDAC4, bioreactor 3D cell culture, mechanobiolgy, interpenetrating polymer network, tissue engineering

## Abstract

The limited ability of articular cartilage to self-repair has motivated the development of tissue engineering strategies that aim to harness the regenerative potential of mesenchymal stem/marrow stromal cells (MSCs). Understanding how environmental factors regulate the phenotype of MSCs will be central to unlocking their regenerative potential. The biophysical environment is known to regulate the phenotype of stem cells, with factors such as substrate stiffness and externally applied mechanical loads known to regulate chondrogenesis of MSCs. In particular, hydrostatic pressure (HP) has been shown to play a key role in the development and maintenance of articular cartilage. Using a collagen-alginate interpenetrating network (IPN) hydrogel as a model system to tune matrix stiffness, this study sought to investigate how HP and substrate stiffness interact to regulate chondrogenesis of MSCs. If applied during early chondrogenesis in soft IPN hydrogels, HP was found to downregulate the expression of *ACAN, COL2, CDH2* and *COLX*, but to increase the expression of the osteogenic factors *RUNX2* and *COL1*. This correlated with a reduction in SMAD 2/3, HDAC4 nuclear localization and the expression of NCAD. It was also associated with a reduction in cell volume, an increase in the average distance between MSCs in the hydrogels and a decrease in their tendency to form aggregates. In contrast, the delayed application of HP to MSCs grown in soft hydrogels was associated with increased cellular volume and aggregation and the maintenance of a chondrogenic phenotype. Together these findings demonstrate how tailoring the stiffness and the timing of HP exposure can be leveraged to regulate chondrogenesis of MSCs and opens alternative avenues for developmentally inspired strategies for cartilage tissue regeneration.

## Introduction

The avascular, aneural and alymphatic nature of cartilage tissue hinders its ability to self-repair, leading to progressive joint damage following injury (Bernhard and Vunjak-Novakovic, 2016). To date, neither conventional cartilage repair treatments such as microfracture or autografting, or regeneration strategies such as autologous chondrocytes implantation (ACI) (Brittberg et al., 1994) can predictably restore the damaged tissue to its original state. Major limitations of these applications include donor-site morbidity, lack of integration and dedifferentiation of chondrocytes during *in vitro* expansion (Huey et al., 2012; Moran et al., 2014; Mumme et al., 2016). Tissue engineering strategies that aim to recapitulate aspects of mesenchymal condensation and cartilage development represent promising new approaches for joint regeneration (Lalan et al., 2001; Bernhard and Vunjak-Novakovic, 2016; Occhetta et al., 2018). Developmentally, cartilage formation begins with mesenchymal condensation leading to chondrogenic differentiation of mesenchymal cells (Wu et al., 2013). In response to cell condensation, a dense matrix is produced, serving as a cartilage anlage, which will lead to the formation of both articular cartilage and subchondral bone (Liu et al., 2017). In the context of a developmentally inspired strategy for cartilage tissue engineering, mesenchymal stem/stromal cells (MSCs) represent a promising cell source due to their ease of isolation and expansion and capacity to give rise to different musculoskeletal tissues (Mardones et al., 2015; Occhetta et al., 2018). MSC differentiation depends on cues present within the local environment, and while much attention has focused on soluble factors to direct their chondrogenic differentiation, less attention has been given to physical stimuli such as substrate rigidity and external mechanical forces (Kelly and Jacobs, 2010; Thorpe et al., 2010; Steward and Kelly, 2014; O'Reilly and Kelly, 2016; Foyt et al., 2019).

Lineage commitment of MSCs can be regulated by the elasticity of the substrate and its topography (Engler et al., 2006; Guilak et al., 2009; Jaalouk and Lammerding, 2009; Huebsch et al., 2010; Romanazzo et al., 2012; Murphy et al., 2014; Foyt et al., 2018). These studies have typically explored the role of substrate stiffness on MSC fate in 2D culture systems (Evans et al., 2009; Holle and Engler, 2011; Evans and Gentleman, 2014), however the role of matrix stiffness in directing differentiation in a 3D hydrogel environment is more complex, with factors such as hydrogel degradation also playing a role (Khetan et al., 2013). In general, higher hydrogel stiffnesses have been shown to promote osteogenesis, while adipogenesis is supported when their stiffness decreases (Huebsch et al., 2010). In the context of chondrogenesis, a stiffness mimicking the rigidity of healthy articular cartilage (0.5 MPa) has been shown to enhance the expression of *SOX9, ACAN* and *COL2* in primary chondrocytes and ATDC5 cells (a chondrogenic cell line) grown on 2D substrates (Allen et al., 2012). However, chondrogenesis of human MSCs has been shown to be supported by much softer substrates (~1 kPa) (Park et al., 2011), with factors such as the local oxygen tension also influencing cellular response to altered substrate rigidity (Foyt et al., 2019).

In addition to matrix stiffness, other biophysical stimuli such as compression and hydrostatic pressure (HP) have also been shown to regulate chondrogenesis of stem cells (Kelly and Jacobs, 2010; Steward and Kelly, 2014). *In vivo*, the removal of physical cues has been associated with the arrest of embryogenesis (Martin et al., 2009; Behrndt et al., 2012). Thus, in order to drive development, mechanical signals must be presented in an appropriate spatial and temporal manner in combination with biochemical cues (Kumar et al., 2017). Hydrostatic pressure, a loading modality that results in little or no cellular deformation, is a key regulator of chondrogenesis (Pattappa et al., 2019). HP has been shown to play a role in regulating chondrogenic differentiation during limb development, while the application of physiological frequencies and magnitudes of HP (up to 12 MPa) promotes an increase of cartilage matrix synthesis and regulates hypertrophy (Soltz and Ateshian, 2000; Angele et al., 2003; Carter and Wong, 2003; Elder and Athanasiou, 2009; Huey et al., 2012; Giorgi et al., 2014; Saha et al., 2016; Pattappa et al., 2019). In the presence or absence of exogenous TGF-β, cyclic HP has been shown to enhance chondrogenesis of MSCs (Miyanishi et al., 2006; Vinardell et al., 2012a; Carroll et al., 2013; Zellner et al., 2015), with the cellular response to such signals also depending on the stiffness of the surrounding substrate (Steward et al., 2012a, 2014b). Understanding the interplay between matrix stiffness and extrinsic mechanical cues such as HP will be critical to the development of tissue engineering strategies aiming to use MSCs to generate phenotypically stable articular cartilage.

Despite HP emerging as a key mechanical stimuli for the development and maintenance of articular cartilage, tissue engineering strategies using MSCs that successfully integrate this regulatory cue have yet to be established (Elder and Athanasiou, 2009). The goal of this study was to investigate how HP, substrate stiffness and TGF-β3 interact to regulate chondrogenesis of MSCs. To this end, this study examined the combined effect of HP and substrate stiffness in the presence of TGF-β3 on the chondrogenic commitment of MSCs seeded within collagen/alginate interpenetrating networks (IPN) hydrogels (Gillette et al., 2008, 2010; Branco da Cunha et al., 2014). The initial hypothesis of this study was that HP combined with TGF-β3 stimulation would enhance chondrogenesis of MSCs embedded in a soft 3D IPN, while it would help rescue the chondrogenic phenotype in MSCs seeded in the stiffer matrix. This study found that the early application of HP inhibited the condensation of MSCs and suppressed the expression of key chondrogenic markers. In contrast, the delayed application of HP promoted cellular condensation and the maintenance of a chondrogenic phenotype in MSCs maintained in a soft hydrogel. These findings demonstrated how diverse biophysical cues can be integrated to regulate chondrogenesis of MSCs and open alternative avenues for developmentally inspired strategies for cartilage tissue regeneration.

## Materials and Methods

### Experimental Design

This study was designed to examine the role of HP and substrate stiffness on the chondrogenic differentiation of MSCs in a 3D culture model. Initially, MSCs seeded IPNs were stimulated with cyclic HP for 7 days in presence of chondrogenic medium containing TGF-β3. In a second set of studies, 3D IPNs were first cultured for 7 days in chondrogenic medium (containing TGF-β3), followed by 1 week of culture in chondrogenic medium and HP stimulation. All study groups consisted of a hydrostatically loaded group (HP) and a free-swelling unloaded control (FS).

### Cell Isolation and Culture

Porcine bone marrow-derived MSC were isolated from the femora of 4 months old porcine donors (50 kg) within 2 h of sacrifice and expanded in culture. Following colonies formation, MSCs were trypsinized, counted and seeded at a density of 5.000 cells cm^−2^ in culture flasks (Nunclon; Nunc, VWR) maintained in growth medium (GM) composed of high-glucose Dulbecco's modified eagles medium (hgDMEM Glutamax) supplemented with 10% v/v fetal bovine serum (FBS), penicillin/streptomycin (100 U ml^−1^) (all GIBCO, Invitrogen) and expanded to passage 3 in a humidified atmosphere at 37°C and 5% CO_2_. Trilineage Potential assays were used to determine pluripotency and one donor was selected (Supplementary Figure 1). For differentiation studies, MSC were supplemented with chondrogenic differentiation media (CDM) composed of hgDMEM, penicillin/streptomycin (100 U ml^−1^), 100 μg ml^−1^ sodium pyruvate, 40 μg ml^−1^ L–proline, 1.5 mg ml^−1^ bovine serum albumin, 4.7 g μl^−1^ linoleic acid, 1X insulin-transferrin-selenium, 50 μg ml^−1^ L–ascorbic acid−2– phosphate (all Sigma–Aldrich), 100 nM dexamethasone (Sigma–Aldrich) and 10 ng ml^−1^ TGF–β3 (R&D Systems). Cells were cultured in CDM for 7 or 14 days at 5% pO_2_, in a humidified atmosphere at 37°C and 5% CO_2_.

### 3D IPN Fabrication and Culture

The collagen-alginate 3D IPNs were prepared starting by 1 ml of 6 mg ml^−1^ ice-cold collagen type I solution (from rat tail, Corning), which had the latter addition of 400 μl of 10 × RPMI (Sigma–Aldrich) and 350 μl of collagen neutralization buffer (0.1 M HEPES and 1 M sodium bicarbonate dissolved in PBS) to reach pH 7.4. At this point, a volume of 400 μl of MSCs previously trypsinized and resuspended in GM (5 × 10^−6^ cells ml^−1^), was gently mixed to the neutralized collagen solution. Finally, 2 ml of 3.5% alginate solution (UP LVG, batch# BP-0907-02, viscosity = 198 mPa^*^s, Pronova matrix) were thoroughly mixed, pipetted into a custom-made mold and placed in incubation for 4 h to allow cell spreading before the addition of the crosslinker (20 mM CaCl_2_ dissolved in hgDMED). Finally, the elasticity of the IPNs was tuned by exposing the samples to the crosslinker for either 40 min (soft 3D IPNs) or 150 min (stiff 3D IPNs). After the incubation time, the 3D IPNs samples were removed from the mold, rinsed in fresh hgDMEM and incubated in GM for 12 h at 5% pO_2_, in a humidified atmosphere at 37°C and 5% CO_2_.

### Application of Cyclic Hydrostatic Pressure

**Figure 1:**
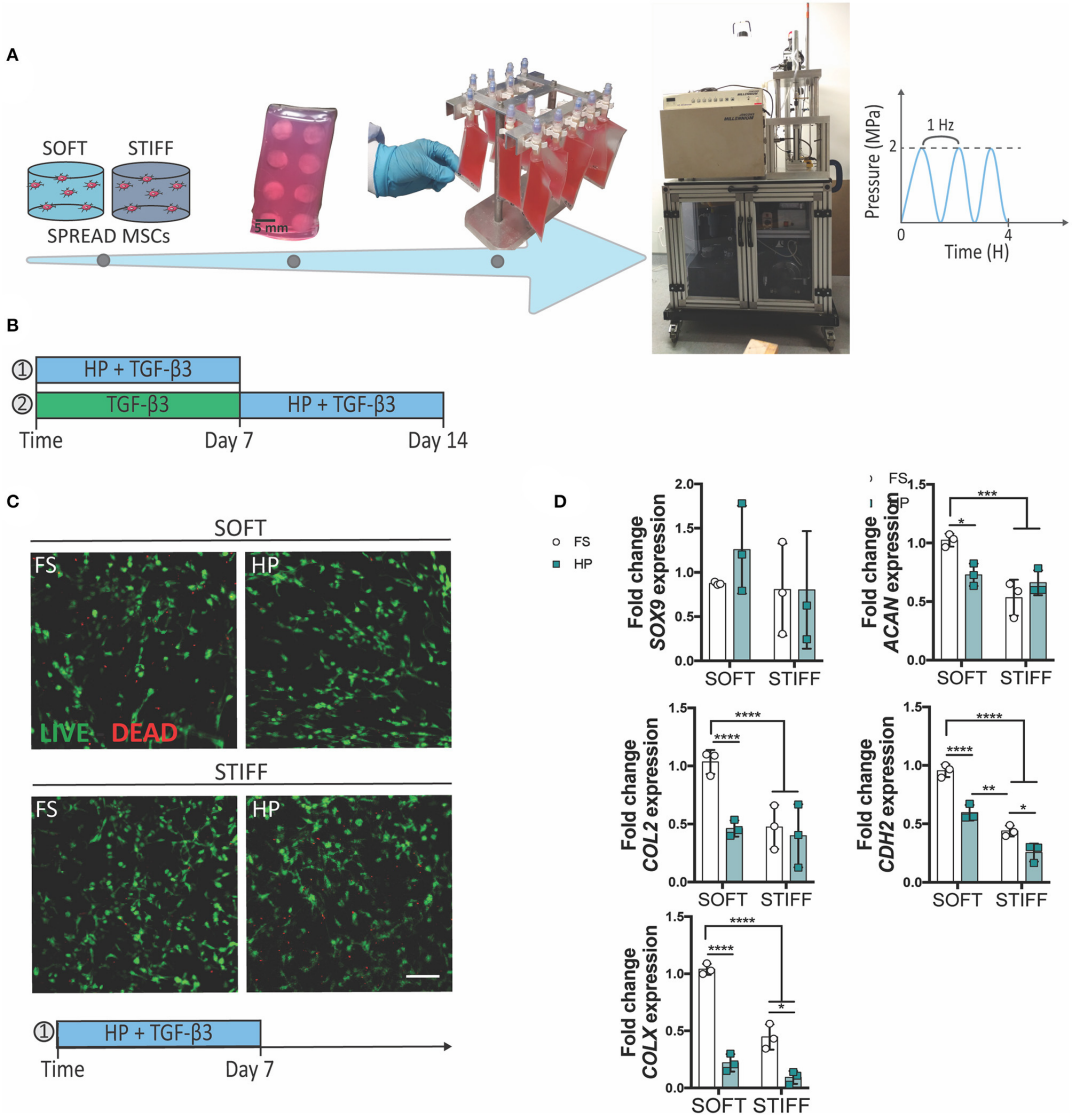
The inhibitory effect of early HP stimulation on chondrogenesis of MSCs. Schematics depicting **(A)** the sample preparation procedure and **(B)** the experimental design. **(C)** Qualitative analysis of MSC viability after 7 days of dynamic culture. Live cells stained in green, dead cells in red. Scale bar 100 μm. **(D)** Chondrogenic gene expression levels relative to the SOFT FS group. MSCs were cultured for 7 days in presence of chondrogenic factors and HP stimulation. FS, Free Swelling group; HP, Hydrostatic Pressure group. *p < 0.05, **p < 0.01, ***p < 0.001, ****p < 0.0001.

The schematic in Figure 1A shows the preparation of the samples for the HP loading experiments. Cell-laden IPN hydrogels were prepared and left to equilibrate overnight in GM. The next day, the IPN samples were encapsulated, evenly spaced, in a 2% agarose (A2790, Sigma–Aldrich) block to provide protection from handling damage and inserted into heat-sealed, gas-permeable, water-tight, sterile bags (EVO120, Quest Biomedical, UK) with 3 mL of medium per construct, removing the air via a needle free port. Before the loading experiment, samples were incubated for 24 h in starvation media [hgDMEM, penicillin/streptomycin (100 U/mL) and 0.5% FBS (Gibco)] for cell cycle synchronization. Cyclic HP was applied via a water filled, custom-made bioreactor within a 37°C incubator as described previously (Carroll et al., 2013). The sealed bags exposed to HP were placed into the pressure vessel, while the free swelling controls were placed into an open water bath next to the pressure vessel. HP was applied at an amplitude of 2 MPa and a frequency of 1 Hz for a duration of 4 h per day, for 7 consecutive days (or 14 days when specified). The bags were returned to a culture incubator (37°C, 5% CO_2_, 5% pO_2_) between loading periods and suspended separately in an upright position for homogenous gas transfer.

### Live/Dead Assay

Viability of MSCs embedded into IPN hydrogels was investigated before the media change to CDM or at the end of the culture period by using a live/dead assay solution consisting of Calcein-AM (1 mM) and Propidium Iodide (0.1 mM) prepared in phenol-red free DMEM (hgDMEM, GIBCO). Briefly, samples were rinsed in PBS, immersed in the staining solution for 1 h at 37°C. Then, samples were rinsed twice with phenol-red free hgDMEM and left in warm phenol-red free hgDMEM before imaging. Live imaging was performed using a confocal microscope (Leica Confocal Microscopy TCS SP8) using 490/515 nm (excitation/emission) for Calcein-AM (Life Technologies) and 535/617 nm (excitation/emission) for propidium iodide. Maximum intensity projection images were obtained using FIJI software.

### Immunostaining

Immediately after HP stimulation/culture period, samples were fixed with 4% PFA by direct injection of the solution into the bags. Samples were left for 1 h at +4°C. Gels were then rinsed in PBS, removed from the culture bag and the agarose block discarded. The retrieved gels were next incubated overnight in 30% w/v sucrose solution at +4°C. The samples were then placed in a mix of 50% v/v of a 30% w/v sucrose solution, and 50% v/v OCT (Tissue-Tek) for 5 h at +4°C. Finally, the samples were placed in OCT and frozen in a isopentane (Sigma–Aldrich) bath previously chilled in liquid nitrogen and stored at −80°C. Sections of 40 μm were cut with a cryostat (Leyca CM 1860) and mounted on glass slides (VWR) previously custom-coated with gelatin (Sigma-Aldrich), left to dry for 30 min and stored at −20°C until stained. Cryoslices were left to re-equilibrate at room temperature for 5 min while single slices boundaries were drawn with a pap-pen (Ted Pella). Samples were permeabilized in 0.5% v/v Triton-X-100 (Sigma–Aldrich), rinsed once in PBS and incubated in blocking buffer containing 5% w/v BSA (Sigma–Aldrich) before the incubation with the primary and secondary antibodies (Table 1 for complete list of reagents). Samples were mounted with 2.5 μl of ProLong gold antifade (Thermo Fisher scientific) on each slice of sample, covered with a coverslip (VWR) and left to cure for 1 h at room temperature and then overnight at +4°C.

**Table 1:** List of antibodies and toxins.

**PRIMARIES**			
Rabbit polyclonal anti-NCAD	1:150	Abcam	ab18203
Rabbit polyclonal anti-SMAD 2/3	1:200	Santa Cruz	sc8332
Rabbit monoclonal anti-VIMENTIN	1:500	Abcam	ab92547
Rabbit polyclonal anti-HDAC4	1:200	Santa Cruz	sc11418
**SECONDARIES**			
Goat anti-rabbit Alexa-488	1:500	Thermo Fisher	a11008
Goat anti-mouse Alexa-488	1:500	Thermo Fisher	a21202
Goat anti-mouse Alexa-594	1:500	Thermo Fisher	a21203
Phalloidin-iFluor 647-cytopainter	1:1500	Thermo Fisher	ab176759
DAPI	0.1 μg ml^−1^	Thermo Fisher	D1306

### RNA Isolation and Gene Expression Analysis

The gels were washed once in PBS, mechanically digested and then incubated with 1 ml of alginate dissolving buffer (0.055 M sodium citrate, in 0.03 EDTA, 0.15 M NaCl, pH 6.8 (all Sigma–Aldrich) for 10 min at 37°C. Samples were then centrifuged for 2 min at 10,000 rpm, rinsed in PBS and centrifuged for 2 min at 14,500 rpm. The resulting pellets were lysed with 350 μl of RLT buffer containing 1% β-Mercaptoethanol with the latter addition of 540 μl of RNA-free water and 10 μl of Protease K solution (Qiagen) and kept for 10 min at 55°C. Total RNA was extracted using the RNeasy Mini Kit (Qiagen) following manufacturer instructions, snap frozen in liquid nitrogen and stored at−80°C. Polymerase chain reaction (PCR) with a high capacity cDNA reverse transcription kit (Thermofisher) was conducted to transcribe 300 ng of RNA from each sample into cDNA. After cDNA quantification with Qubit ssDNA Assay kit (Thermofisher), levels of gene expression were measured with real-time PCR (ABI 7500-fast, Applied Biosystems) using SYBR green master mix (Applied Biosystems) and porcine specific primers (Table 2). The relative quantity of each sample was calculated with the Pfaffl (2001) method with reference to 18 S and B2M and expressed as fold change to the control group (specified in each figure legend). Efficiency of all primer pairs were calculated by serial dilutions of cDNA reverse transcribed from RNA isolated from day 7 porcine MSC pellets cultured in CDM.

**Table 2:** List of specific primers for real time PCR.

**Gene name**	**Gene full name**	**Forward/Reverse**	**T(^**°**^C) in use**	**T(^**°**^C) predicted**	**Gene ID**	**Gen bank no**.
*B2M*	Beta-2-microglobulin	F:5′ ACTGAGTTCACTCCTAACG 3′	60	54.2	397033	NM_213978
		R:5′ TGCAGCATCTTCATAATCTC 3′		58		
*18S*	Ribosomal protein S18	F:5′ CAACACCACATGAGCATATC 3′	60	59	396980	NM_213940
		R:5′ AGAAGTTCCAGCACATTTTG 3′		59.4		
*ACAN*	Aggrecan	F:5′ CACCCCATGCAATTTGAG 3′	60	62.6	397255	NM_001164652
		R:5′ AGATCATCACCACACAGTC 3′		55.5		
*SOX9*	SRY-box 9	F:5′ GACTGCTGAATGAGAGCGAGA 3′	60	59.87	396840	NM_213843.2
		R:5′ GAAGATGGCGTTGGGAGAGAT 3′		59.86		
*CDH2*	Cadherin 2	F:5′ AGGTTTGCCAGTGTGACTCC 3′	60	60.18	100515322	XM_021096205.1
		R:5′ TCTCGGCGCTTCATCCATAC 3′		59.97		
*COL1*	Collagen type I alpha 1 chain	F:5′ TAGACATGTTCAGCTTTGTG 3′	60	56.4	100738123	XM_021067153
		R:5′ GTGGGATGTCTTCTTCTTG 3′		57.1		
*COL2*	Collagen type II alpha 1 chain	F:5′ CGACGACATAATCTGTGAAG 3′	60	58.3	397323	XM_001925959
		R:5′ TCCTTTGGGTCCTACAATATC 3′		59.3		
*COLX*	Collagen type X alpha 1 chain	F:5′ CCAACATCCAGAATCCATC 3′	60	60.04	448809	NM_001005153
		R:5′ GTAGGTGTTTGGTATTGCTC 3′		59.3		
*RUNX2*	Runt related transcription factor 2	F:5′ CCAACAGAGGCATTTAAGG 3′	60	59.7	100155806	XM_003482202
		R:5′ CCAAAAGAAGTTTTGCTGAC 3′		59		

### Image Quantification

Samples were imaged the day after the staining with a Leica SP8 scanning confocal microscope (equipped with lasers for 405, 488, 552, and 638 nm and 3PMT detectors) with X 10, 20, or X 40 (1.3 numerical aperture, oil-immersion) objective lens. Z-stack images were acquired with an interval of 1 μm, using the same exposure, gain and offset values for all conditions in the same experiment. Pictures were taken from three random areas of each sample. These parameters were set based on positive controls expressing the protein of interest, and negative controls obtained by omitting the primary antibody. SMAD 2/3, HDAC 4 nuclear quantification and NCAD cytosolic quantification were calculated using a custom-made code for FIJI 3D Image Suite for the 3D mask generation, followed by the formula:

$$\begin{array}{l} \sum_{nuc}^{f}/V_{nuc}\;or\;\sum_{cyto}^{f}/V_{cyto} \end{array}$$

Where $\overset{f}{\underset{nuc}{\sum }}\;$and $\overset{f}{\underset{cyto}{\sum }}\;$represent the sum of the background-corrected intensity values for the voxels in the nuclear and cytoplasmic region respectively, while *V*_*nuc*_ and *V*_*cyto*_ the volume of the corresponding regions.

### Statistical Analysis

Statistical analyses were performed on three independent trials using one- or two-way analysis of variance (ANOVA) followed by Tukey *post-hoc* test [GraphPad Prism 6.0 statistical software (GraphPad Software)]. Significance was accepted at a level of p < 0.05. Numerical and graphical results are presented as mean ± standard deviation.

## Results

### The Early Application of Cyclic Hydrostatic Pressure Inhibits the Chondrogenic Commitment of MSCs

To understand the role of HP in regulating the initiation and progression of chondrogenesis, MSCs were encapsulated within soft (5.2 ± 0.7 kPa) or stiff (17.5 ± 1.8 kPa) 3D IPN hydrogels and subjected to HP and TGF-β3 (10 ng/ml within the culture media) stimulation. In agreement with our previous findings, the softer 3D IPNs were more supportive of MSC chondrogenic differentiation. To facilitate construct handling during the loading phase, the 3D IPN samples were embedded into agarose blocks, placed into cell-culture bags and loaded at a frequency of 1 Hz for 4 h/day at 2 MPa (Figure 1A). A first set of studies were conducted in presence of TGF-β3 for 7 days (Figure 1B), while in the second set of studies MSCs were first cultured for one week in presence of TGF-β3, followed by a week of HP stimulation in presence of the same chondrogenic factor (Figure 1B). Sample handling and HP loading didn't cause any detrimental effect on cell viability (Figure 1C, day 7 +HP+TGF-β3). In free swelling conditions, higher expression of *ACAN, COL2, CDH2* and *COLX* were observed in the softer hydrogels after 1 week of culture (Figure 1D). However, the application of TGF-β3 + HP stimulation reduced the expression of *ACAN, COL2, CDH2* and *COLX* when MSCs were encapsulated within the softer IPN hydrogels (Figure 1D). In the stiffer hydrogels, HP reduced the expression of *CDH2* and *COLX* (Figure 1D). The already low levels of *ACAN* and *COL2* expression in the stiff hydrogels were not further reduced by the application of HP.

**Figure 2:**
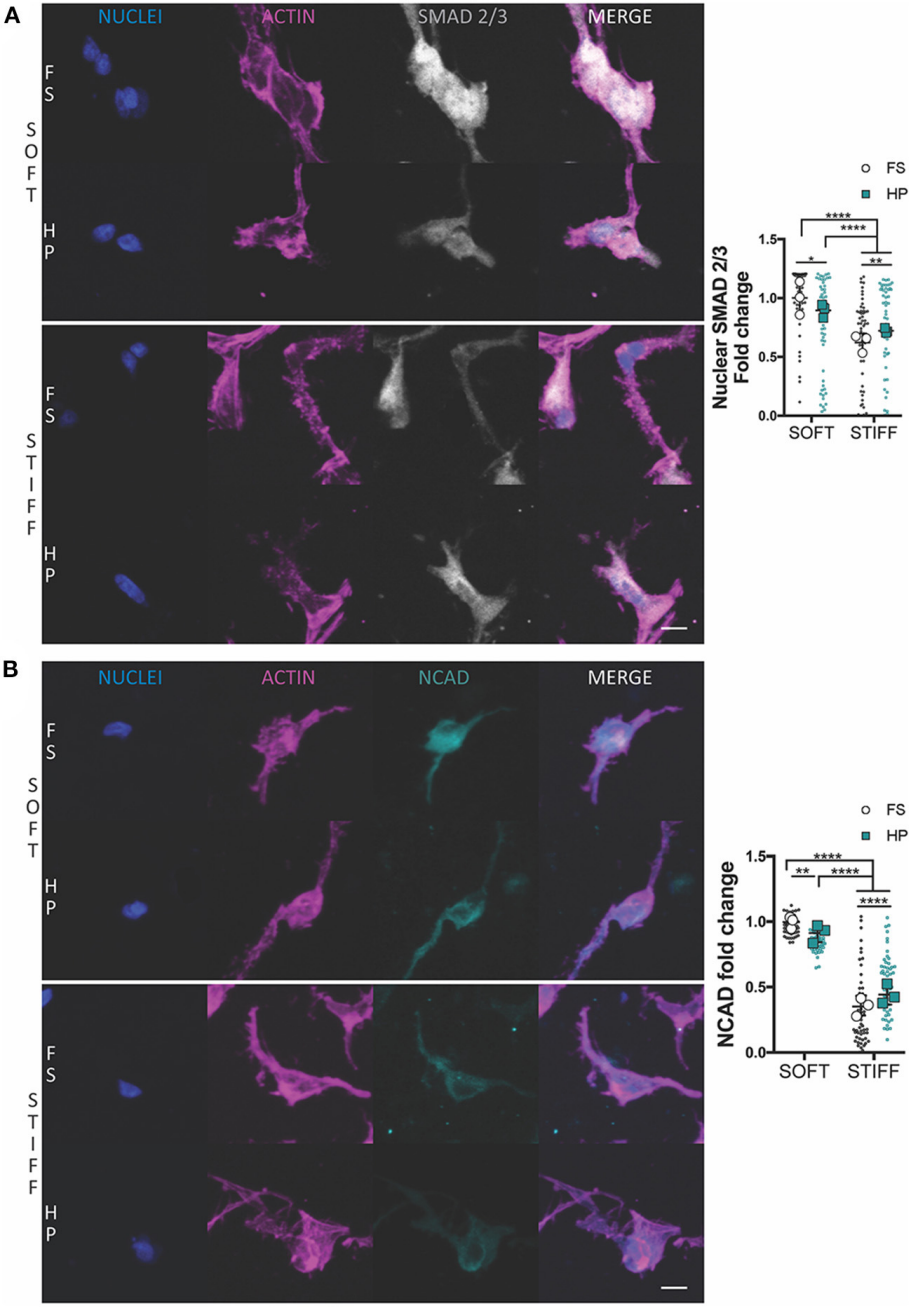
The influence of HP on nuclear SMAD 2/3 and NCAD levels in MSCs cultured within soft and stiff IPN hydrogels. Confocal analysis of MSCs cultured for 7 days in presence of TGF-β3+HP. Cells stained for nuclei (blue), actin (magenta) and **(A)** SMAD 2/3 (gray) or **(B)** NCAD (cyan). Scale bars, 10 μm. Quantitative analysis of confocal images to determine the fold change of **(A)** the nuclear content of SMAD 2/3 and **(B)** NCAD expression. *p < 0.05, **p < 0.01, ****p < 0.0001.

The suppression of chondrogenesis following the immediate application of HP to the soft hydrogels correlated with a reduction in the nuclear levels of SMAD 2/3 (Figure 2A) and a minor reduction in NCAD expression (Figure 2B). An opposite effect was observed in the stiffer hydrogels, where the application of HP was observed to increase nuclear SMAD 2/3 levels (Figure 2A) and total NCAD (Figure 2B) secretion, although the overall levels of both proteins remained lower when compared to the soft hydrogels.

### Early HP Stimulation Suppresses MSCs Condensation and Is Associated With Osteogenesis and Cytosolic HDAC4 Localization

**Figure 3:**
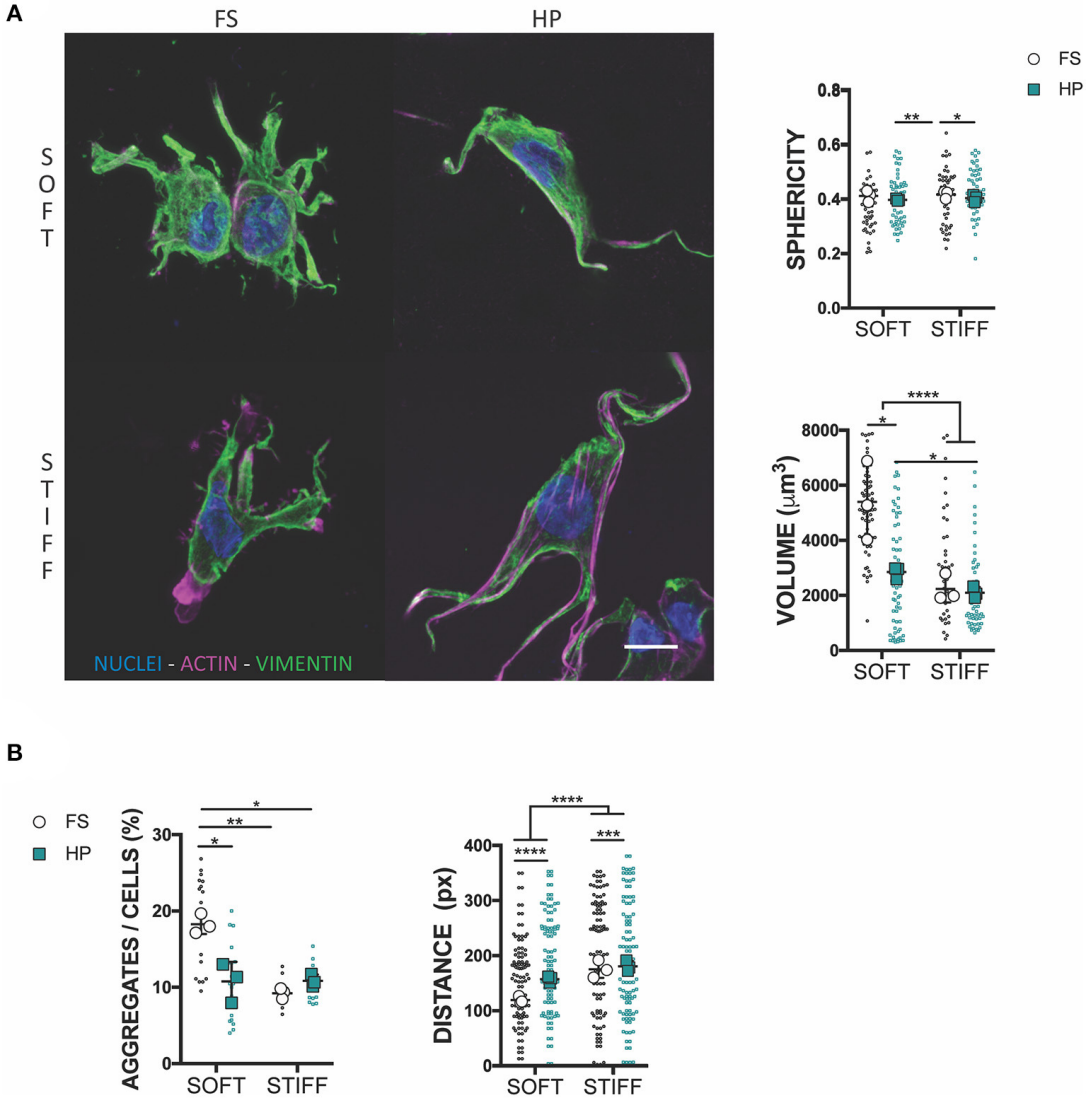
HP stimulation inhibits MSCs aggregation. **(A)** Confocal analysis of MSCs cultured for 7 days in presence of TGF-β3 and HP. Cells stained for nuclei (blue), actin (magenta) and vimentin (green). Scale bar, 10 μm. Graphs reporting the values of sphericity and volume of single cells embedded into 3D IPN. **(B)** Average percentage of cell aggregates per field of view after 7 days of culture and cell-cell distance. *p < 0.05, **p < 0.01, ***p < 0.001, ****p < 0.0001.

To understand the mechanism of HP mechanotransduction, this study next sought to analyze morphological changes in MSCs cultured in soft and stiff hydrogel environments and exposed to TGF-β3 and HP. The intermediate filament architecture, in particular the vimentin network of MSCs, has been shown to remodel under the action of HP (Steward et al., 2012a; Stavenschi and Hoey, 2019). Although assessed only qualitatively, vimentin seemed to form a less interlaced network when exposed to HP and this correlated with a decreased cell sphericity (Figure 3A). Surprisingly, the volume of cells seeded into a soft 3D IPN reduced significantly under HP stimulation, while such changes in cell size were not observed in the stiffer hydrogels (Figure 3A). Furthermore, following the application of HP cell aggregation in the soft matrix was impaired, while no evident effect on cellular aggregation was observed in the stiffer 3D IPN (Figure 3B). Although HP didn't significantly affect the tendency of cells to aggregate/condense in the stiffer 3D IPN, the average distance between single cells increased in both soft and stiff hydrogels after HP stimulation (Figure 3B).

**Figure 4:**
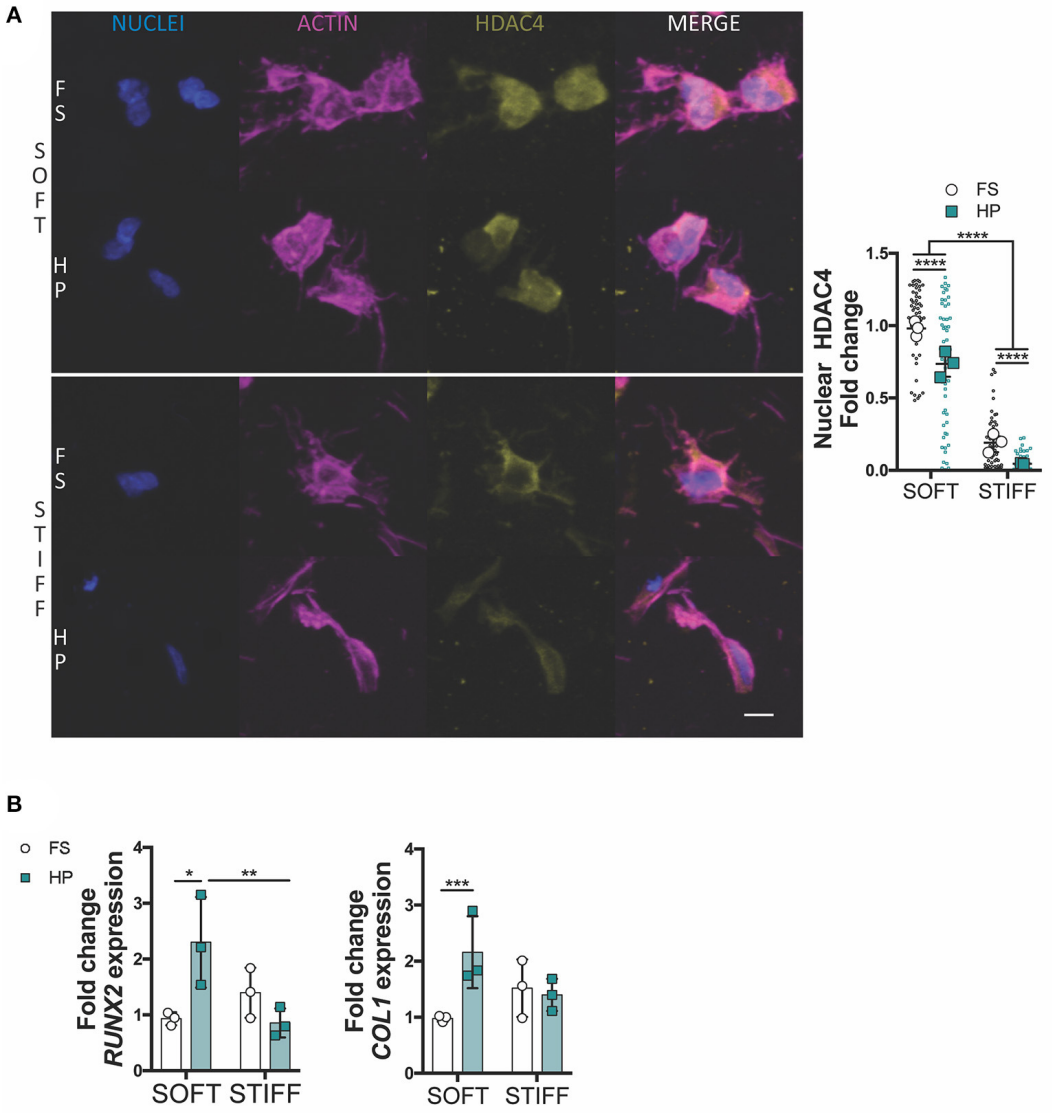
HDAC4 nuclear localization in MSCs is responsive to HP stimulation. **(A)** Evaluation of HDAC4 localization and nuclear content. Cells stained for nuclei (blue), actin (magenta) and HDAC4 (yellow). Scale bar, 10 μm. **(B)** Day 7 gene expression relative to SOFT FS group. *p < 0.05, **p < 0.01, ***p < 0.001, ****p < 0.0001.

To further investigate how HP impaired MSCs chondrogenesis, the expression of key osteogenic and hypertrophic markers was next studied. HDAC4 is known to be a potent regulator of chondrocyte hypertrophy by inhibiting *RUNX2* expression (Pei et al., 2009; Studer et al., 2012). Nuclear HDAC4 levels were higher in soft gels compared to stiff gels. The early application of HP was observed to reduce HDAC4 nuclear localization in MSCs encapsulated in both soft and stiff hydrogels (Figure 4A). Furthermore, the application of HP enhanced the expression of *RUNX2* and *COL1* in the soft hydrogels, while no significant effect was observed in the stiffer gels (Figure 4B).

### A Delayed Exposure to HP Enhances Chondrogenesis of MSCs

**Figure 5:**
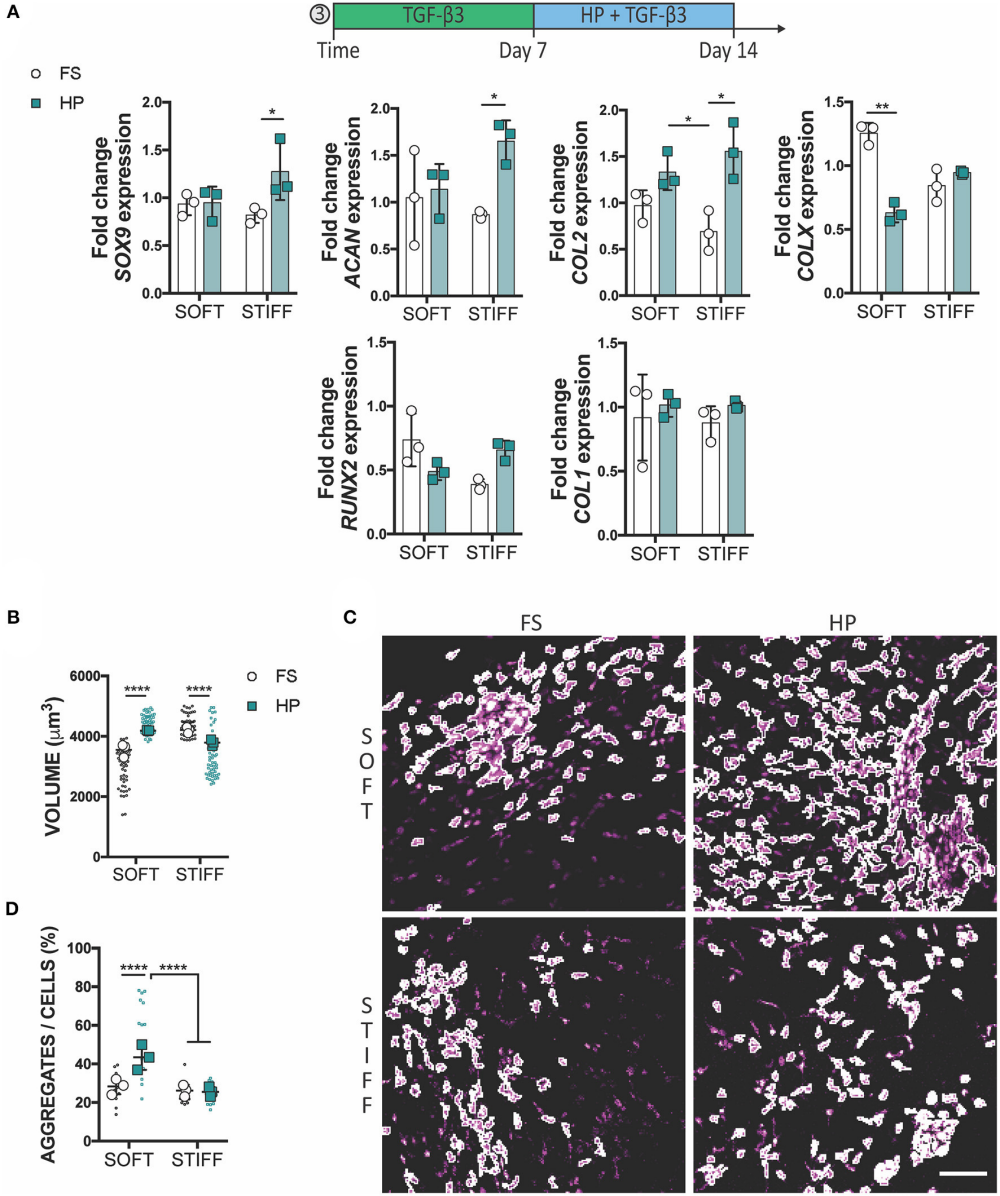
Delayed exposure to HP enhances chondrogenesis. **(A)** Gene expression analysis of MSCs cultured for 1 week in presence of TGF-β3 followed by 1 week of HP and TGF-β3. **(B)** Graphs reporting single cell volume after 14 days of culture. **(C)** Representative images of cell aggregates and **(D)** their quantification. Cells stained for actin (magenta). Scale bar 100 μm, *p < 0.05, **p < 0.01, ****p < 0.0001.

To assess whether a delayed exposure to HP would enhance the chondrogenic differentiation of MSCs, cells were cultured for 1 week in free swelling chondrogenic conditions (+TGF-β3), followed by a week of HP stimulation in the same media conditions. In this case, the application of HP had no (positive or detrimental) effect on the expression of *SOX9, ACAN* and *COL2* in MSCs encapsulated within the soft 3D IPN, although it did suppress the expression of *COLX*. In contrast, HP enhanced the expression of *SOX9, ACAN*, and *COL2* in MSCs encapsulated within the stiffer matrix which was previously found to be less supportive of a chondrogenic phenotype (Figure 5A). From a morphological standpoint, HP promoted cell volume expansion in the softer 3D IPN, but not in the stiffer hydrogel (Figure 5B). Surprisingly, an increase in cell aggregation with the application of delayed HP was only observed in the softer matrix (Figures 5C,D).

## Discussion

It has been previously demonstrated by our lab that physiological levels of cyclic HP stimulation have beneficial effects on the long term chondrogenic commitment of bone marrow derived MSCs (Meyer et al., 2011; Carroll et al., 2013; Steward et al., 2014b). However, the mechanism by which matrix stiffness and HP interact to regulate MSC commitment remains poorly understood. This study confirms that a biomaterial able to facilitate cell-cell interactions and aggregation over time creates a supportive environment for MSC chondrogenesis, and that applying cyclic HP one week after the initiation of chondrogenesis can further enhance this process and promote a more stable cartilage phenotype. In contrast, the application of HP at the onset of MSC differentiation inhibited chondrogenesis and promoted a more osteogenic phenotype. In these studies, early HP stimulation was associated with vimentin remodeling, cell volume restriction and a reduced degree of cell condensation. In contrast, when MSCs were allowed to first initiate chondrogenic differentiation in free swelling conditions (i.e., one week of TGF-β3 supplementation), the application of HP no longer suppressed chondrogenesis. Indeed, such a delayed mechanical stimulation was found to enhance cell aggregation, suppress markers of hypertrophy in the soft 3D IPN and enhance the expression of chondrogenic genes in the stiff hydrogels. These findings highlight that cyclic HP can directly modulate MSC fate in a manner that depends on substrate stiffness and timing of HP exposure. Altogether these studies provide a novel platform for MSCs differentiation analysis and may open new possibilities to develop loaded-assisted cartilage tissue engineering strategies.

The early application of HP, combined with TGF-β3 supplementation, was associated with a decrease in cell aggregation and a downregulation in chondrogenic markers within the soft 3D IPN. This correlated with a reduction in the nuclear levels of SMAD 2/3 and a reduced production of NCAD. *In vitro*, MSCs chondrogenic differentiation is elicited by cell condensation, which is mediated by NCAD (Kwon et al., 2018). However, as cells become chondrogenic, the expression of NCAD decreases (Kwon et al., 2018). Cells undergoing condensation activate the SMAD2/3 complex, which translocates to the nuclear compartment where it regulates *SOX9* expression, which in turn controls the transcription of the major cartilage matrix proteins *COL2* and *ACAN* (Woods et al., 2007). In this study, the application of HP reduced the mRNA levels of *ACAN, COL2* and *COLX* in MSCs maintained in a soft matrix to levels comparable to that in a stiffer, unloaded, environment. The early application of HP to the stiffer 3D IPN, which is an inherently less chondro-supportive environment, was unable to trigger a more robust chondrogenic response, despite increases in SMAD2/3 nuclear localization.

Both matrix stiffness and HP were observed to play a role in vimentin remodeling and the morphology of the encapsulated MSCs. It is know that vimentin architecture changes upon HP stimulation, both in 2D and 3D cell culture models (Steward et al., 2012b; Stavenschi and Hoey, 2019), and it has been hypothesized that HP could induce its depolymerisation(Steward et al., 2012b; Pattappa et al., 2019). *In vivo*, vimentin increases cytoplasmic elasticity and plays a role in the alignment of cell traction forces needed for directed mesenchymal migration; from rheology measurements, its network is known to be easily deformable and able to withstand high strains without breaking (Janmey et al., 1991; Guo et al., 2013; Costigliola et al., 2017). The early application of HP was also found to reduce cellular volume in the soft hydrogel environment. Cellular size control has been shown to be a robust modulator of MSC and chondrocyte fate. MSC volume expansion has been correlated to osteogenic differentiation, whilst chondrocyte confinement is associated with reduced secretion of cartilage matrix proteins and the promotion of a more catabolic phenotype (Lee et al., 2017, 2019). The results of this study suggest a possible interplay between vimentin pressure-induced changes and cell volume adaptation, although further studies are required to firmly established such a link. A deeper understanding of the interdependency of vimentin remodeling, cell volume adaptation and cell migratory behavior would be of benefit in developing loading-induced MSC differentiation strategies.

The nuclear localization of HDAC4 was found to depend on both substrate stiffness and hydrostatic pressure, suggesting HDAC4 shuttling during chondrogenesis is sensitive to such mechanical cues. It is known that mechanical perturbations can alter the state of the nucleus and in some cases physical signals reach the nucleus before soluble cues (Wang et al., 2009; Aragona et al., 2015; Driscoll et al., 2015). For instance, fluid flow induced shear stress has been shown to modulate chromatin condensation and increase nuclear stiffness in endothelial cells (Deguchi et al., 2005). Twisting the cytoskeleton *via* magnetic beads has been shown to cause direct force transmission to the nucleus and elicit local chromatin remodeling (Iyer et al., 2012). Histone deacetylases (HDACs) participate in epigenetic regulation by keeping the chromatin in a highly packed form, wrapped around histones (Haberland et al., 2009). In particular, HDAC4 is a potent regulator of chondrocytes hypertrophy and its nuclear transport is initiated by TGF-β through the activation of SMADs complexes (Vega et al., 2004; Studer et al., 2012; Wang et al., 2014). Subcellular relocation of HDAC4 can be modulated by physical signals; indeed compressive loading of chondrocytes has been shown to induce HDAC4 nuclear import and gene regulation (Chen et al., 2016). Although the role of HP on HDAC4 shuttling is not clear in the literature, our findings suggest that both a stiffer substrate and early HP exposure might inhibit the nuclear import of HDAC4. HP stimulation was associated with reduced nuclear HDAC4 in MSCs encapsulated within both soft and stiff hydrogels, although increases in *RUNX2* and *COL1* were only observed in the softer hydrogels, confirming the detrimental effect of early HP on chondrogenic differentiation in this model system. It is possible that MSCs grown in the soft 3D IPN might be more sensitive to HDAC4 shuttling than cells experiencing a stiffer environment, which already demonstrated lower levels of nuclear HDAC4. Although these findings linked for the first time the role of substrate stiffness and HP as possible regulators of HDAC4 during MSC chondrogenic differentiation, more investigations would be needed to understand how these physical stimuli interact to control HDAC4 behavior.

The delayed application of HP was associated with increased cellular aggregation for MSCs grown in soft hydrogels and an upregulation of *SOX9, ACAN* and *COL2* in cells encapsulated within stiffer matrices. The delayed application of HP was also found to reduce the expression of *COLX* in MSCs encapsulated within soft 3D hydrogels. At this stage of differentiation (day 14) a reduction of *COLX* may be indicative of this mechanical stimulus suppressing hypertrophy and progression along an endochondral pathway (Caron et al., 2012). The dynamics of cellular condensation within these IPN hydrogels may play a key role in determining the temporal response of chondrogenically primed MSCs to HP. It has been previously shown that MSCs cultured in the form of pellets positively responded to HP stimuli (Miyanishi et al., 2006). MSCs cultured as pellets are forced to aggregate at the onset of chondrogenesis. In contrast, MSCs encapsulated in hydrogels are initially relatively isolated, but in response to TGF-β3 stimulation begin to undergo chondrogenesis. As part of this process, and particularly in the soft IPN that supports robust chondrogenesis, MSCs began to form aggregates within the hydrogel. It is possible that early stimulation with HP might have suppressed MSC aggregation [especially for our low seeding density relative to other 3D hydrogel studies (Wagner et al., 2008; Meyer et al., 2011; Steward et al., 2012a, 2014a; Carroll et al., 2013)], whilst delaying HP to first allow MSCs to condensate might have supported the more beneficial response to this mechanical stimulus that is typically reported in the literature. Interestingly, the delayed application of HP to MSCs maintained in soft matrices promoted cellular volume expansion, while a reduction in cell volume was observed for MSCs kept in the stiffer hydrogels. Previous studies have linked the confinement of chondrocyte volume to an inhibition of cartilage matrix production (Lee et al., 2017). Although not directly examined, it is possible that volume regulation plays a key role in the mechano-transduction of HP, however this hypothesis needs further examination.

A limitation of this study, from a translational perspective, is our use of porcine MSCs as a model system to explore how hydrostatic pressure regulates the initiation and progression of stem cell chondrogenesis. The capacity of porcine MSC to undergo chondrogenesis has been demonstrated in previous studies (Thorpe et al., 2012; Vinardell et al., 2012b; Daly et al., 2016) (see also Supplementary Figure 1). MSCs of porcine origin have been suggested to provide a useful animal model system to evaluate tissue engineering strategies (Ringe et al., 2002), since their genetics, anatomy and physiology are similar to humans (Vacanti et al., 2005). Another advantage of using relatively young, healthy porcine MSCs for such studies is that they typically possess a consistent, predictable phenotype when exposed to differentiation factors, which facilitates the exploration of how biophysical cues such as hydrostatic pressure regulate their fate. Future studies should, however, confirm the findings of this study using human MSCs. A key part of such a study would be to explore the extent of donor-to-donor variability in the response of MSCs to mechanical cues such as HP, which was not undertaken in this study. Finally, an analysis of the long-term phenotypic stability of chondrogenically primed MSCs that are co-stimulated with HP is warranted, as such cues could help promote a stable chondrogenic phenotype.

To conclude, in presence of TGF-β3, the application of 2 MPa of HP for 4 h per day applied at the onset of chondrogenesis (from day 0 to day 7 of culture) generally inhibited cellular condensation and MSC chondrogenesis. In contrast, the application of HP from day 7 to day 14 of culture generally enhanced chondrogenesis of MSCs. Although the supplementation of a supra-physiological level of TGF-β3 is a potent regulator of MSC chondrogenesis, mechanical stimulation can positively or negatively modulate its effect depending on the timing of its application. In the context of cartilage regeneration, this study demonstrates that physical stimuli such as matrix stiffness and HP are fundamental regulators of MSC fate, whose interaction must be carefully considered to successfully engineer functional cartilaginous tissues.

## Data Availability Statement

The raw data supporting the conclusions of this article will be made available by the authors, without undue reservation.

## Ethics Statement

The animal study was reviewed and approved by The Health Products Regulatory Authority in accordance with protocols approved by Trinity College Dublin Animal Research Ethics Committee.

## Author Contributions

DK and PA conceived the study, designed experiments, interpreted results, and wrote the manuscript. PA conducted experiments with intellectual input from DK. All authors have given approval to the final version of the manuscript.

## Conflict of Interest

The authors declare that the research was conducted in the absence of any commercial or financial relationships that could be construed as a potential conflict of interest.
