# Supplementary material for: Hydrostatic Pressure Regulates the Volume, Aggregation and Chondrogenic Differentiation of Bone Marrow Derived Stromal Cells
Source: Front Bioeng Biotechnol. 2021 Jan 15;8:619914. doi: 10.3389/fbioe.2020.619914 (PMC7844310; doi:10.3389/fbioe.2020.619914)
Supplement: Supplementary file 1 [file Table_1.docx]

Supplementary Material

Supplementary 1 | MSCs tripotentiality assessment. MSCs at p3 stained for adipogenesis (Oil Red O, (A), osteogenesis (Alizarin Red, (B) and chondrogenesis (Alcian Blue, C) after 21 days of culture. Scale bars: 5 µm (A and B) and 100 μm (C).

**Effect of HP stimulation on MSCs proliferation.**

Immediately after loading, samples (n ≥ 4) were wased in PBS, the wet mass recorded and stored at -80°C. The samples were mechanically and then enzymatically digested in a papain solution ((125μg/ml) in 0.1 M sodium acetate, 5mM L-cysteine-HCl and 0.05 M EDTA pH 6.0, all Merck) ) at 60°C under constant rotation for 18 hours followed by the addition of 1 M sodium citrate solution for 1 h to dissociate crosslinked alginate. DNA level was detected with the Quant-iT™ PicoGreen® dsDNA Assay Kit (Thermoscientific) following the manufacturer protocol and the results normalized to the dry weight were reported as fold change of day 0 values.

|   Supplementary 2 \| Effect of HP on DNA content. Analysis of the DNA content of MSCs cultured for 7 days in presence of TGF-β and HP. Values from three independent trials and reported as fold change of day 0, indicated by the dotted line. * p<0.05, **p<0,01, & reports p<0.05 respective of day 0 values. |
| --- |
